# Neurological complications following cerebral angiography: a case of contrast-induced encephalopathy

**DOI:** 10.1186/s12883-025-04536-3

**Published:** 2025-12-05

**Authors:** Ai-Hsien Li, Shao-Cheng Lin, Chih-Wei Yao, Pao-Hao Chiu, Shih-Tsung Cheng

**Affiliations:** 1 Health Management Center, Far Eastern Memorial Hospital, No. 21, Section 2, Nanya S. Road, Banqiao District, New Taipei, 220 Taiwan; 2 Present Address: Everan Hospital, Taichung, Taiwan; 3 College of Information, Yuan_ze University, Taoyuan, Taiwan; 4 Department of Internal Medicine, College of Medicine, National Taiwan Univerisity, Taipei, Taiwan

**Keywords:** Contrast-induced encephalopathy, Stroke, Middle cerebral artery, Angiography, Brain edema, Seizure, Blood-Brain barrier, Neurotoxicity

## Abstract

**Background:**

Contrast-induced encephalopathy (CIE) is a rare but increasingly recognized iatrogenic complication associated with the use of intravascular iodinated contrast agents, particularly during neurovascular procedures. It often presents with acute neurological deficits that mimic ischemic strokes or seizure disorders. Despite its often reversible course, radiological and electroclinical mimicry of other acute conditions poses a diagnostic dilemma.

**Case presentation:**

We report the case of an 82-year-old woman with multiple vascular comorbidities who developed acute left hemiparesis and conjugate gaze deviation after diagnostic cerebral angiography. Initial non-contrast CT revealed a dense right middle cerebral artery (MCA) sign, raising concerns about acute large vessel occlusion. However, subsequent angiography revealed no occlusions. Supportive neurocritical care, including hydration and anticonvulsant therapy, resulted in complete neurological recovery within five days. Electroencephalography (EEG) revealed decreased amplitude, indicating focal cortical dysfunction in the right hemisphere without epileptiform activity. Interval imaging confirmed the resolution of right hemispheric edema.

**Conclusion:**

This case underscores the diagnostic challenges of CIE, particularly its ability to radiologically and clinically mimic acute ischemic strokes. The absence of EEG epileptiform discharges, despite convulsive symptoms, highlights the non-epileptic mechanisms underlying contrast-induced neurotoxicity. Enhanced interdisciplinary awareness and radiologic vigilance are essential to prevent unnecessary interventions and improve patient outcomes.

## Introduction

Contrast-induced encephalopathy (CIE) is a rare but potentially debilitating neurological complication that occurs following intra-arterial administration of iodinated contrast media, particularly in neurovascular and coronary interventions. Clinically, it may manifest as acute encephalopathy, seizures, cortical blindness, hemiparesis, or other focal deficits, often mimicking acute cerebrovascular syndromes such as ischemic stroke or subarachnoid hemorrhage [1, 2]. Although typically reversible within 72 h, CIE can occasionally result in irreversible injuries or fatal outcomes [3, 4].

From a pathophysiological standpoint, the leading hypothesis involves disruption of the blood–brain barrier (BBB), allowing leakage of contrast into the parenchymal or subarachnoid spaces and inducing vasogenic edema [5, 6]. However, experimental and clinical evidence increasingly supports alternative or concurrent mechanisms, including the direct neurotoxicity of iodinated agents, particularly in patients with renal dysfunction or preexisting microangiopathy [7, 8].

Key risk factors include chronic hypertension, diabetes mellitus, chronic kidney disease, prior cerebrovascular disease, and the use of high-dose or hyperosmolar contrast media [9–11]. Radiologically, CIE may present with cortical or subcortical hyperdensities on CT, gyriform or sulcal enhancement on MRI, and focal edema without diffusion restriction, distinguishing it from infarction [12, 13]. Electroencephalography (EEG) abnormalities, when present, are often nonspecific, typically showing focal slowing without epileptiform discharges, further complicating the clinical differentiation [14, 15].

Despite the growing awareness, CIE remains underdiagnosed in many countries. Management remains supportive, focusing on volume expansion, seizure control, and reducing secondary injury. No consensus exists regarding pharmacologic prophylaxis or treatment [16–18].

## Case presentation

An 82-year-old woman with a significant vascular history of hypertension (treated with amlodipine/valsartan), type 2 diabetes mellitus (on metformin), prior ischemic stroke (residual left hemiplegia; aspirin since 2017), and bilateral varicose veins presented with episodic dizziness suggestive of posterior circulation insufficiency. Ancillary findings included a history of cataracts, insomnia, and chronic constipation.

Carotid duplex ultrasonography revealed mild-to-moderate atherosclerotic stenosis at both bifurcations, and Vertebral Doppler ultrasonography revealed hypoplasia of the right vertebral artery (diameter, 1.7 mm; flow, 6 mL/min) with compensatory flow in the left vertebral system. These findings support the diagnosis of vertebrobasilar insufficiency. Diagnostic digital subtraction angiography was performed.

Angiographic findings included:

- 30% stenosis of the left ICA bulb.
- 55% stenosis of the petrous ICA segment.
- 70% stenosis of the right MCA M2 segment.
- 60% ostial stenosis of the left vertebral artery.
- Hypoplasia of the right vertebral artery.
- Coronary angiography (concurrent): 50% stenosis of the PDA; 70% stenosis at the LAD origin.

**Fig. 1:**
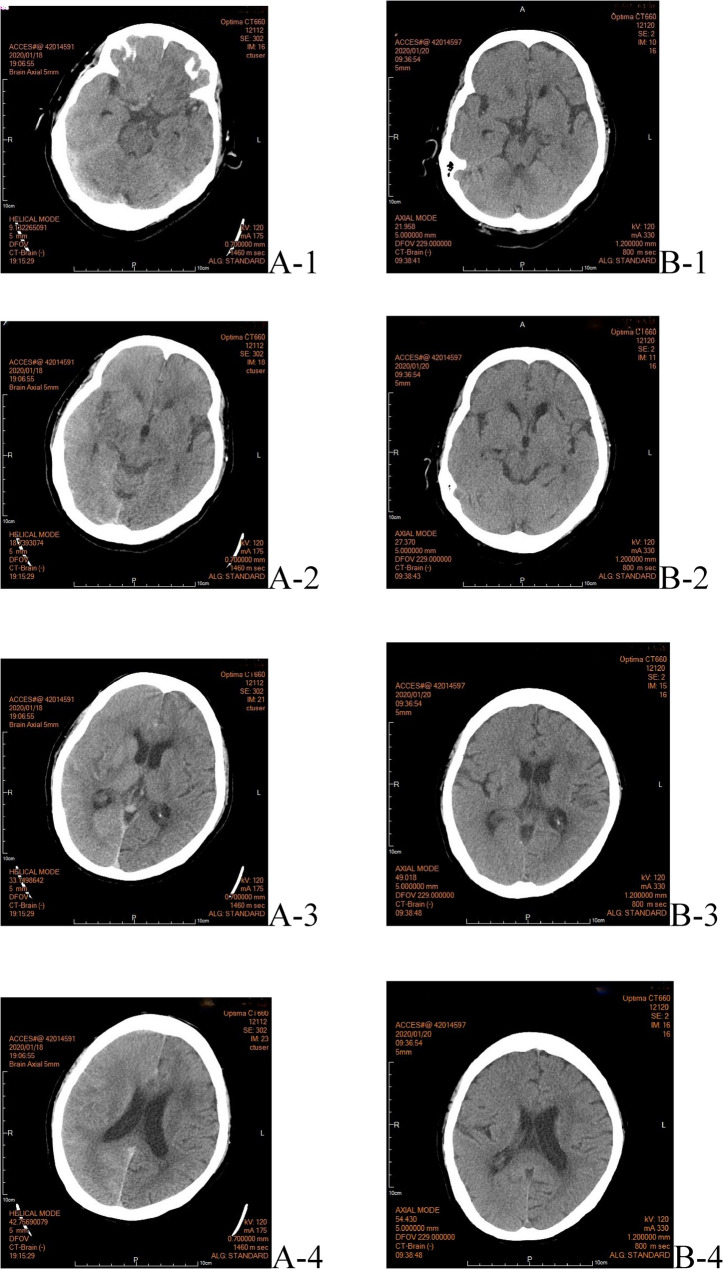
Serial non-contrast CT brain scan. **A** Immediate post-procedure CT reveals a hyperdense right MCA sign (arrow in A-1) and diffuse right hemispheric ‘white-out’ with effacement of the sulci (A-2, A-3,A-4). **B** Follow-up CT at 48 h demonstrated complete resolution of the hyperdensity and edema, with reappearance of normal sulcal patterns (B-2, B-3,B4). A-1 (CIE-1) at the pons level: right side dense MCA sign (+), diminished right side sulcus, and blurred gray-white matter junction with enhanced brightness over the right hemisphere. A-2 (CIE-2) at 3rd ventricle level/basal cisterna: diminished right-side sulcus and blurred gray-white matter junction with enhanced brightness over the right hemisphere. A-3 (CIE-3) at the lateral ventricle with anterior and posterior horns: disappearance of the right-sided sulcus and a blurred gray-white matter junction with enhanced brightness over the right hemisphere. A-4 (CIE-4) at the falx/genu and splenium of the Corpus Callosum level: showing disappearance of the right-side sulcus and blurred grey-white matter junction with enhanced brightness over the right hemisphere, as well as some linear hyperdensity over earlier sulci. Panel **B** Follow-up CT (48 h later) showing resolved edema.B-1 (PCIE-1) at the pons level as A-1: reappearance of some sulci with a still blurred gray-white matter junction. B-2 (PCIE-2) at 3rd ventricle level/basal cisterna as A-2: re-appearance of some Sulci, with more prominent Sylvian fissure and disappearance of right hemi-sphere “white-out” B-3 (PCIE-3) at the lateral ventricle with anterior and posterior horns as A-3: reappearance of some sulci of the frontal lobe with a still blurred gray-white matter junction. B-4 (PCIE-4) at Falx and genu and splenium of Corpus Callosum level: re-appearance of some Sulci over right hemisphere (still less than left side) (Generally speaking , right hemisphere diffuse “ white-out” is clearly appreciated over A-2, A-3, and A-4, and it disappeared in B-2, B-3, and B-4.)

**Fig. 2:**
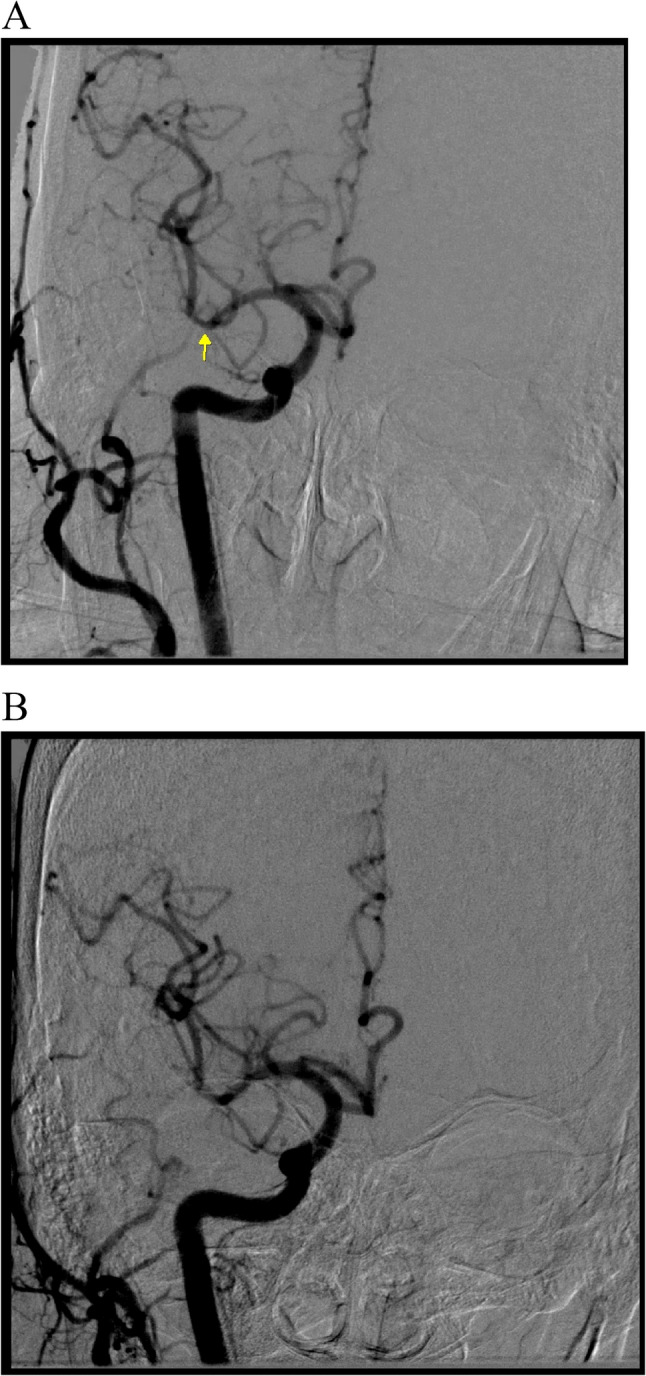
Angiography confirmed TICI 3 flow without occlusion (arrow). **A** initial right common carotid angiography showed a distal middle cerebral artery stenosis (arrow) with TICI 3 flow. **B** Repeated angiography showed similar findings with TICI 3 flow

Within 2 h post-procedure, the patient experienced an acute right hemispheric headache, hypertensive surge, left hemiparesis (MRC grade 2/5), conjugate gaze deviation to the right, and reduced responsiveness. Non-contrast brain CT revealed a dense right middle cerebral artery (MCA) sign and subtle sulcal effacement, radiographically suggesting an acute thromboembolic stroke (Fig. 1A). In addition, right hemisphere “white-out” with diminished sulci and blurred gray-white matter interphase were also noted. However, urgent reangiography revealed a patent MCA flow (TICI 3) with no evidence of thromboembolism (Fig. 2).

The patient was admitted to the neurological ICU. Intravenous hydration with isotonic saline was initiated for the patient. Antiplatelet therapy was changed from aspirin to clopidogrel due to the risk of gastrointestinal bleeding (hemoglobin 7.4 g/dL; suspected stress-related ulceration). Twelve hours later, she developed a generalized tonic-clonic seizure that required intubation and treatment with Levetiracetam.

**Fig. 3:**
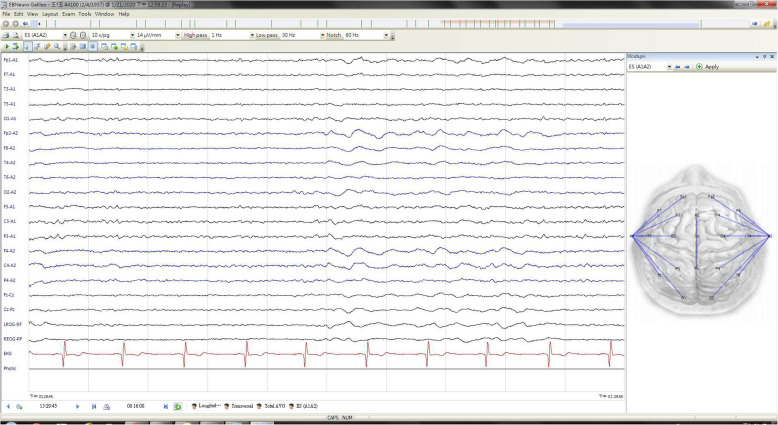
EEG on the 4th day: The study showed decreased amplitude over the right hemisphere (red bracket), indicative of focal cortical dysfunction. No epileptiform discharge was observed

Repeat CT (Fig. 1B) on day 2 showed resolution of sulcal effacement and disappearance of the hyperdense MCA sign. No infarction or hemorrhage was observed. Electroencephalography (EEG) on day 4 revealed decreased amplitude in the right hemisphere, indicating focal cortical dysfunction without epileptiform activity.(Fig. 3) The patient’s creatinine level transiently rose to 1.32 mg/dL (eGFR 38 mL/min/1.73 m²), suggestive of mild contrast-induced nephropathy. MRI was not performed during acute hospitalization because of the patient’s acute instability requiring intubation and sedation following seizure management.

The patient also developed aspiration pneumonia and received antibiotics and red blood cell transfusions, and by day five, the patient’s neurological status returned to baseline. The patient was successfully extubated after the procedure. No residual neurological deficits were observed at the time of discharge.

## Discussion

This case exemplifies the profound diagnostic challenge posed by CIE, primarily because of its ability to mimic acute ischemic stroke. The initial non-contrast CT finding of a dense MCA sign, a classic radiological hallmark of thromboembolic occlusion, was the most compelling red herring in our patient [12, 13]. However, the subsequent confirmation of patent vasculature and the rapid resolution of radiological signs confirmed that this was a false positive, representing contrast pooling within the vascular compartment or the parenchyma due to severe blood-brain barrier (BBB) disruption [5, 19]. MRI was not performed during acute hospitalization because of the patient’s acute instability requiring intubation and sedation following seizure management. However, the combination of rapid imaging resolution and complete neurological recovery with supportive care supported the diagnosis of CIE without the need for advanced MRI characterization.

The pathophysiological mechanism of CIE, characterized by transient BBB failure leading to vasogenic edema and direct neurotoxicity, is crucial for understanding the clinical and radiological manifestations of CIE. The absence of epileptiform discharges on EEG, despite the occurrence of convulsions, suggests a non-epileptic mechanism, potentially involving transient cortical irritation or metabolic dysfunction induced by contrast agent extravasation [14, 15, 20]. Although the patient’s EEG did not exhibit epileptiform activity, this does not definitively rule out the possibility of subclinical or transient epileptic events. It is important to recognize that electrical abnormalities may remain undetected if they are intermittent or localized. The patient experienced a tonic-clonic seizure, indicating the presence of epileptiform activity during the acute phase. Furthermore, the patient’s EEG demonstrated decreased amplitude, suggesting focal cortical dysfunction in the right hemisphere. This evidence supports the evolving perspective that CIE encompasses a spectrum of neurotoxic responses that are independent of conventional ictogenesis.

Despite the use of iso-osmolar contrast agents, the patient’s marginal renal function, cumulative contrast exposure, and pre-existing cerebrovascular susceptibility rendered her prone to contrast-induced encephalopathy (CIE) [9, 21–23]. Advanced imaging modalities, such as dual-energy computed tomography (CT) or iodine mapping, may further assist in distinguishing contrast staining from genuine ischemia or hemorrhage. Additionally, the initially concerning dense middle cerebral artery (MCA) signs highlighted the possibility of acute MCA embolic occlusion immediately following diagnostic angiography. However, literature exists that addresses the potential for false positives associated with this well-recognized sign [24].

Notably, no pharmacological agents have been definitively proven to prevent or reverse CIE. While corticosteroids and osmotic diuretics are employed empirically, their efficacy remains uncertain [17, 18]. In light of the pathophysiology of CIE, transient contrast-induced vasoconstriction followed by proximal MCA congestion and direct neuronal injury due to temporary disruption of the blood-brain barrier (BBB), accompanied by brain edema, have been proposed [25]. Consequently, the literature has reported the potentially therapeutic role of corticosteroids, although our patient improved without their use [9, 14, 15]. This patient responded well to supportive measures alone—hydration, seizure control, and careful monitoring—highlighting the importance of early recognition to avoid unnecessary thrombolytic and surgical interventions in the future.

Research on the predilection sites within the vascular territories of CIE remains limited. However, some scholars have hypothesized that vascular territories experiencing chronic ischemia or pre-existing severe cerebral vasculopathies may be more prone to contrast-related BBB leakage, which can subsequently lead to CIE [11, 14]. This hypothesis may explain the diffuse “white-out” observed in our patient’s right MCA territory, which had a pre-existing stenotic lesion (Fig. 2). Additionally, Allison et al. proposed that the lateralization of CIE across various vascular territories might be linked to the localized injection of a contrast bolus [15]. This case highlights the critical importance of cautious contrast administration in patients with pre-existing vascular lesions to prevent CIE.

## Conclusion

CIE remains a diagnostic challenge, particularly in patients with overlapping vascular risk profiles and ambiguous radiological findings. Neurologists and radiologists must maintain a high index of suspicion when evaluating acute neurological deterioration following angiography. Recognition of characteristic clinical-imaging dissociation and EEG findings can prevent misdiagnoses. Comprehensive supportive care is the cornerstone of its management. Further research is required to elucidate the predictive factors and improve the use of contrast agents in vulnerable populations.

## Data Availability

All data generated or analyzed in this study are included in this article.

## Abbreviations

CIE: Contrast-induced encephalopathy
EEG: electroencephalography
